# Structure aware Runge–Kutta time stepping for spacetime tents

**DOI:** 10.1007/s42985-020-00020-4

**Published:** 2020-07-28

**Authors:** Jay Gopalakrishnan, Joachim Schöberl, Christoph Wintersteiger

**Affiliations:** 1 Portland State University, PO Box 751, Portland, OR 97207 USA; 2 Technische Universität Wien, Wiedner Hauptstraße 8-10, 1040 Wien, Austria

**Keywords:** Local time stepping, Spacetime, Causality, 65M60, 65M20

## Abstract

We introduce a new class of Runge–Kutta type methods suitable for time stepping to propagate hyperbolic solutions within tent-shaped spacetime regions. Unlike standard Runge–Kutta methods, the new methods yield expected convergence properties when standard high order spatial (discontinuous Galerkin) discretizations are used. After presenting a derivation of nonstandard order conditions for these methods, we show numerical examples of nonlinear hyperbolic systems to demonstrate the optimal convergence rates. We also report on the discrete stability properties of these methods applied to linear hyperbolic equations.

## Introduction

For simulating wave phenomena, the state of the art relies heavily on efficient and accurate numerical solution techniques for hyperbolic systems. This paper is concerned with those solution techniques that proceed by subdividing the spacetime into tent-shaped subregions satisfying a causality condition. Just as light cones are often used to delineate what is causally possible and impossible in the spacetime, tent-shaped spacetime regions are natural to impose causality when numerically solving hyperbolic equations. By constraining the height of the tent pole, erected vertically in an increasing time direction, one can ensure that the tent encloses the domain of dependence of all its points. This constraint on the tent pole height is a causality condition that a numerical scheme using such tents should satisfy. The spacetime subdivision into tents may be unstructured, thus allowing such schemes to advance in time by different amounts at different spatial locations, i.e., local time stepping can be naturally built in while subdividing the spacetime into tents.

The main contribution of this paper is a new explicit Runge–Kutta type time stepping scheme for solving hyperbolic systems within a spacetime tent. Standard time stepping methods cannot be directly applied on tents, since tents are generally not a tensor product of a spatial domain with a time interval. A non-tensor product spacetime tent can be mapped to a tensor product spacetime cylinder using a degenerate Duffy-like transformation. This is the basis of the Mapped Tent Pitching (MTP) schemes that we introduced previously in Ref. [5]. As shown there, a spacetime Piola map can be used to pull back the hyperbolic system from the tent to the spacetime cylinder. Being a tensor product domain, the spacetime cylinder, admits the use of standard explicit time stepping schemes, like the classical RK4 scheme. However, as we shall show here, expected convergence rates are *not* observed when such standard explicit schemes are used. The cause of this problem can be traced back to the degeneracy of the map. After illustrating this problem, we shall introduce a new Structure Aware Runge–Kutta (SARK) scheme, which overcomes this problem.

We first reported the above-mentioned order reduction in [6], where a fix was proposed for *linear* hyperbolic systems, called the Structure Aware Taylor (SAT) scheme. In contrast, the new SARK schemes of this paper are applicable to *both linear and nonlinear* hyperbolic systems.

Prior work on tent-based methods spans both the computational engineering literature [2, 11] and numerical analysis literature [4, 12]. These works have clearly articulated the promise of tent-based schemes, including local time stepping, even with higher order spatio-temporal discretizations, and the opportunities to utilize concurrency. Recent advances include tent-based Trefftz methods [13] and the use of asynchronous SDG (spacetime discontinuous Galerkin) methods to new engineering applications [1]. One can find explicit methods for conservation laws in the literature, like the finite volume approach in [10], which incorporate local time stepping without the usage of a tent mesh. In contrast to the presented MTP schemes, the extension to high order is yet to be done for those methods.

To place the present contribution in the perspective of these existing works, a few words regarding our focus on explicit time stepping are in order. The ratio of memory movements to flops is very low for explicit schemes, making them highly suitable for the newly emerging many-core processors. However, before the introduction of MTP schemes in Ref. [5], it was not clear that such advantages of explicit time stepping could be brought to any tent-based method. Now that we have an algorithmic avenue to perform explicit time stepping within tent-based schemes, we turn to the study of accuracy and convergence orders. Having encountered the unexpected roadblock of the above-mentioned convergence order reduction, we have been focusing on developing time stepping techniques to overcome it. This paper is an outgrowth of these studies.

In the next section, we quickly review the construction of MTP schemes, showing how the main system of ordinary differential equations (ODEs) that is the subject of this paper arises. In Sect. 3, we show why one should not use standard Runge–Kutta schemes for solving this ODE system. Then, in Sect. 4, we propose our new SARK schemes for solving the ODE system. Sect. 5 derives order conditions for these schemes. In Sect. 6, we study the discrete stability of SARK schemes. Section 7 reports on the good performance of the new schemes when applied to some standard nonlinear hyperbolic systems.

## Construction of mapped tent pitching schemes

**Fig. 1:**
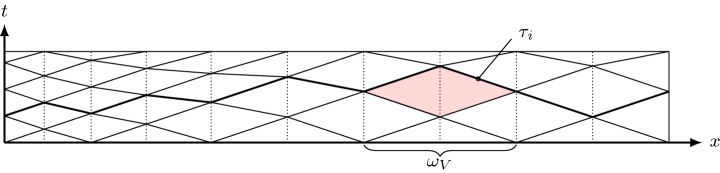
Time slab in one spatial dimension with a local refinement at the left boundary, the advancing front $\tau _i$ at the *i*th step and tent $K_i$ (red) pitched at vertex *V* (color figure online)

In this section we give a brief overview of MTP schemes. Let ${\mathcal {T}}$ be a simplicial conforming spatial mesh of a bounded spatial domain $\varOmega _0 \subset {\mathbb {R}}^N.$ Spacetime tents are built atop this spatial mesh by vertically erecting tent poles at its vertices in a sequence of steps. At the *i*th step, a tent $K_i$ is added. It takes the form

1 $$\begin{aligned} K_i := \{(x,t) : x\in {\omega _V}, \tau _{i-1}(x)\le t\le \tau _{i}\} \end{aligned}$$

where ${\omega _V}$ is the vertex patch made up of all elements in ${\mathcal {T}}$ connected to a mesh vertex *V*. In (1), the function $\tau _i(x)$ is a continuous function of the spatial coordinate *x* that is piecewise linear with respect to ${\mathcal {T}}$. The graph of $\tau _i$ represents the advancing spacetime front at the *i*th step, so the tent $K_i$ may be thought of as the spacetime domain between these advancing fronts—see Fig. 1. Within $K_i$, the distance the central vertex *V* can advance in time is restricted by the *causality constraint*

2 $$\begin{aligned} |\nabla \tau _i| < \frac{1}{c_{\max }}, \end{aligned}$$

where $c_{\max }$ is an upper bound for the local wavespeed on $K_i$ (clarified further below) of a hyperbolic system under consideration. Such an upper bound is readily computable for linear systems. It is also possible to compute such bounds for some nonlinear systems, including the Euler system (without computing the full solution), as described in Ref. [8]. Once we have $c_{\max }$, there are multiple algorithmic options for generating the advancing fronts $\tau _i$ satisfying (2). One such algorithm is written out in detail in our prior work [5].

The hyperbolic systems we have in mind are general systems with *L* unknowns in *N* spatial dimensions, posed on the spacetime cylinder $\varOmega := \varOmega _0 \times (0,t_{{\mathrm {max}}})$ for some final time $t_{{\mathrm {max}}}$. To recall the standard settings in the literature, we follow [3], and assume we are given some $g:\varOmega \times {\mathbb {R}}^L \rightarrow {\mathbb {R}}^{L}$ and $f:\varOmega \times {\mathbb {R}}^L \rightarrow {\mathbb {R}}^{L\times N}$. The standard hyperbolic problem is to find $u:\varOmega \rightarrow {\mathbb {R}}^{L}$ such that

3 $$\begin{aligned} \partial _tg(x,t,u) + {\mathrm {div}}_xf(x,t,u) = 0 \end{aligned}$$

where $\partial _t = \partial /{\partial t}$ denotes the time derivative and ${\mathrm {div}}_x$ denotes the row-wise divergence operator. We assume that the system (3) is hyperbolic in t-direction, as defined in Ref. [3]. This implies, in particular, that the first order derivatives of components of *g* and *f* exist. These derivatives, together with a direction vector, are then used to form a generalized eigenproblem, whose eigenvalues are real whenever (3) is hyperbolic. The absolute values of these eigenvalues, which depend on the direction vector and *x*, *t*, *u*, give wavespeeds. Let *c*(*x*, *t*, *u*) denote the maximum of these wavespeeds over all directions. We choose the quantity $c_{\max }$ in (2) to be any upper bound for these *c*(*x*, *t*, *u*) for all $(x, t) \in K_i$.

MTP schemes proceed by mapping each of the tents arising above to a spacetime cylinder. To define the mapping, we consider a general tent *K* over any given vertex patch ${\omega _V}$, defined by

4 $$\begin{aligned} K := \{(x,t) : x\in {\omega _V}, \varphi _b(x)\le t\le \varphi _t(x)\}. \end{aligned}$$

The functions $\varphi _b$ and $\varphi _t$ are continuous functions that are piecewise linear on the vertex patch and may be identified as the bottom and top advancing fronts restricted to the vertex patch ${\omega _V}$. To map the tent *K* to the spacetime cylinder ${\hat{K}} := {\omega _V}\times (0,1)$, we define the transformation $\varPhi : {\hat{K}} \rightarrow K$ by $\varPhi (x,\hat{t}) := (x,\varphi (x,\hat{t})),$ where

5 $$\begin{aligned} \varphi (x,\hat{t}) := (1-\hat{t})\varphi _b(x) + \hat{t}\varphi _t(x). \end{aligned}$$

Defining $$F:\varOmega \times {\mathbb {R}}^L \rightarrow {\mathbb {R}}^{L\times (N+1)}$$ by

$$\begin{aligned} F(x,t,u) := [f(x,t,u),g(x,t,u)] \in {\mathbb {R}}^{L\times (N+1)}, \end{aligned}$$

we may write (3) as

$$\begin{aligned} {\mathrm {div}}_{x,t}F(x,t,u) = 0. \end{aligned}$$

The spacetime divergence ${\mathrm {div}}_{x,t}$ is a row-wise operator which applies the spatial derivative to the first *N* components and the temporal derivative to the last component. The well-known Piola transformation of *F*, defined by ${\hat{F}} = \left( \det \varPhi '\right) \left( F\circ \varPhi \right) \left( \varPhi '\right) ^{-T}$ can be simplified after calculating the derivatives of $\varPhi$ to

$$\begin{aligned} {\hat{F}} = \left( F\circ \varPhi \right) \begin{bmatrix} \delta I &{} -\nabla \varphi \\ 0 &{} 1 \end{bmatrix} \end{aligned},$$

where $\delta (x) = \varphi _t(x)-\varphi _b(x)$ and $I\in {\mathbb {R}}^{N\times N}$ is the identity matrix. By the properties of the Piola map, we then immediately have

6 $$\begin{aligned} {\mathrm {div}}_{x,\hat{t}}{\hat{F}}(x,\hat{t},{\hat{u}}) = 0, \end{aligned}$$

with ${\hat{u}} = u\circ \varPhi$ and the spacetime divergence ${\mathrm {div}}_{x,\hat{t}}$ on Hence we use${\hat{K}}$. Finally, as described in Ref. [5], writing ${\hat{F}}$ in terms of *f* and *g*, we find that (6) is equivalent to

7 $$\begin{aligned} \partial _{\hat{t}}\left( g(x,\hat{t},{\hat{u}}) - f(x,\hat{t},{\hat{u}}) \nabla \varphi (\hat{t})\right) + {\mathrm {div}}_x\left( \delta (x)f(x,\hat{t},{\hat{u}})\right) = 0, \end{aligned}$$

which is again a conservation law.

For readability, we omit the spatial variable *x* and pseudo-time $\hat{t}$ from the arguments of functions in (7) and simply write

8 $$\begin{aligned} \partial _{\hat{t}}\left( g({\hat{u}}) - f({\hat{u}}) \nabla \varphi \right) + {\mathrm {div}}_x\left( \delta f({\hat{u}})\right) = 0, \end{aligned}$$

which describes the evolution of ${\hat{u}}$ along pseudo-time from $\hat{t}=0$ to $\hat{t}=1$. Since

$$\begin{aligned} \varphi (x,\hat{t}) = (1-\hat{t})\varphi _b(x) + \hat{t}\varphi _t(x) = \varphi _b(x) + \hat{t}\delta (x), \end{aligned}$$

we may split $g({\hat{u}}) - f({\hat{u}}) \nabla \varphi$ into parts with and without explicit dependence on pseudo-time, allowing us to rewrite (8) as

$$\begin{aligned} \partial _{\hat{t}}\left( \left( g({\hat{u}}) - f({\hat{u}}) \nabla \varphi _b\right) - \hat{t}f({\hat{u}}) \nabla \delta \right) + {\mathrm {div}}_x\left( \delta f({\hat{u}})\right) = 0. \end{aligned}$$

This equation is the starting point for our spatial discretization. We use a discontinuous Galerkin method based on

$$\begin{aligned} V_h = \{ v: v|_K \text { is a polynomial of degree } \le p \text { on all spatial elements } T \in {\mathcal {T}}\}. \end{aligned}$$

When restricted to the vertex patch ${\omega _V}$ we obtain $V_h({\omega _V}) = \{ v|_{{\omega _V}}: v \in V_h\}$. Multiplying (8) by a test function $v_h \in V_h$ and integrating by parts over the patch ${\omega _V}$, we obtain

9 $$\begin{aligned} \int _{\omega _V}\frac{ d}{ d{\hat{t}}} \bigg (g({\hat{u}})&- f({\hat{u}}) \nabla \varphi \bigg ) \cdot v_h\nonumber \\&= \sum _{T\subset {\omega _V}}\int _T \delta f({\hat{u}}) : \nabla v_h - \sum _{F\subset {\omega _V}}\int _F \delta f_n({\hat{u}}^+,{\hat{u}}^-) \cdot \llbracket v_h \rrbracket , \end{aligned}$$

for all $v_h\in V_h$ and all $\hat{t}\in [0,1]$. Here and throughout, every facet *F* is assigned a unit normal, simply denoted by *n*, whose direction is arbitrarily fixed, except when $F \subset \partial \varOmega$, in which case it points outward. The traces ${\hat{u}}^+$ and ${\hat{u}}^-$ of ${\hat{u}}$ from either side are defined by

$$\begin{aligned} {\hat{u}}^+ := \lim _{s\rightarrow 0^+}{\hat{u}}(x+sn) \quad \text {and} \quad {\hat{u}}^- := \lim _{s\rightarrow 0^+}{\hat{u}}(x-sn). \end{aligned}$$

In (9), we also used a numerical flux $f_n$ on each facet *F* (that takes values in ${\mathbb {R}}^L$ depending on values ${\hat{u}}^+,{\hat{u}}^-$ from either side) and the jump $\llbracket {\hat{v}}_h \rrbracket := {\hat{v}}_h^+ - {\hat{v}}_h^-$. In these definitions, whenever $\hat{u}^+$ falls outside $\varOmega$, it is prescribed using some given boundary conditions.

Let $m = \dim V_h({\omega _V})$ and let $\psi _i$, $i=1, \ldots , m$ denote any standard local basis for $V_h({\omega _V})$. Introducing $U: [0,1] \rightarrow {\mathbb {R}}^m$, consider the basis expansion

10 $$\begin{aligned} {\hat{u}} (x, \hat{t}) = \sum _{i=1}^m U_i(\hat{t}) \psi _i(x). \end{aligned}$$

Equation (9) leads to an ODE system for $U(\hat{t})$ as follows. Define (possibly nonlinear) operators $M_0: {\mathbb {R}}^m \rightarrow V_h({{\omega _V}})$, $M_1: {\mathbb {R}}^m \rightarrow V_h({{\omega _V}})$, and $A : {\mathbb {R}}^m \rightarrow V_h({{\omega _V}})$ by

11a $$\begin{aligned} \int _{{\omega _V}}M_0(U) v_h&= \int _{{\omega _V}}\left( g({\hat{u}}) - f({\hat{u}}) \nabla _{\!x}\varphi _b\right) \cdot v_h \end{aligned}$$

11b $$\begin{aligned} \int _{{\omega _V}}M_1(U) v_h&= \int _{{\omega _V}}f({\hat{u}}) \nabla _{\!x}\delta \; \cdot \,v_h\end{aligned}$$

11c $$\begin{aligned} \int _{{\omega _V}}A(U) v_h&= \sum _{T\subset {\omega _V}}\int _T \delta f({\hat{u}}) : \nabla v_h - \sum _{F\subset {\omega _V}}\int _F \delta \, f_n({\hat{u}}^+,{\hat{u}}^-) \cdot \llbracket v_h \rrbracket , \end{aligned}$$

for all $v_h \in V_h({{\omega _V}})$, where ${\hat{u}}$ in all terms on the right hand sides above is to be expanded in terms of *U* using (10).

With these notations, problem (9) becomes the problem of finding $U: [0,1] \rightarrow {\mathbb {R}}^m$ satisfying

12 $$\begin{aligned} \frac{d}{d\hat{t}} M(\hat{t}, U(\hat{t})) = A (U(\hat{t})), \end{aligned}$$

given some $U(0) = U_0 \in {\mathbb {R}}^m.$ Here $M : [0, 1] \times {\mathbb {R}}^m \rightarrow V_h({\omega _V})$ is defined by

13 $$\begin{aligned} M(\hat{t}, W) = M_0(W) - \hat{t}M_1(W), \qquad 0 \le {\hat{t}} \le 1, \quad W \in {\mathbb {R}}^m. \end{aligned}$$

## Difficulty with standard time stepping

In this section, we describe the problem we must overcome, thus setting the stage for the new schemes proposed in Sect. 4. The problem is that standard Runge–Kutta methods when applied to the tent system (12)—after a standard reformulation—do not give expected orders of convergence.

A standard approach to numerically solve (12) proceeds by introducing a new variable $Y(\hat{t}) := M(\hat{t}, U(\hat{t})).$ Then, using the inverse mapping $M^{-1}({\hat{t}}, \cdot )$, the primary variable is $U({\hat{t}}) = M^{-1}(t, Y({\hat{t}}))$. Substituting this expression for *U* on the right hand side of (12), we can bring (12) to the standard form

14 $$\begin{aligned} \frac{d}{d\hat{t}}Y(\hat{t}) - A(M^{-1}({\hat{t}}, Y(\hat{t}))) = 0. \end{aligned}$$

Standard ODE solvers, such as the classical explicit RK4 method, may now be directly applied to (14). Unfortunately, this leads to reduced convergence order, as we shall now see.

**Fig. 2:**
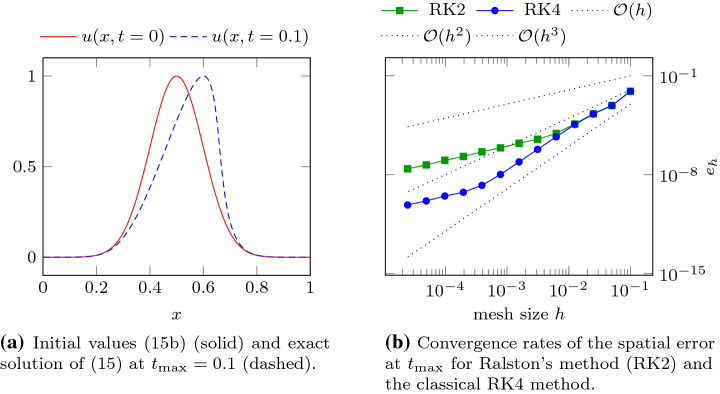
Exact solution *u* and convergence rates of the error $e_h$, defined in (15c), for the example of the Burgers’ equation described in (15)

Consider the example of the one-dimensional Burgers’ equation

15a $$\begin{aligned} \partial _{t} u(x,t) + \partial _x u(x,t)^2 = 0, \quad \forall (x,t)\in [0,1]\times (0,t_{\max }], \end{aligned}$$

with initial values set by

15b $$\begin{aligned} u(x,0)=\exp {\big (-50\big (x-\tfrac{1}{2}\big )^2\big )}, \quad \forall x\in [0,1], \end{aligned}$$

an inflow boundary condition an $x=0$, and an outflow boundary condition at $x=1$. Let $u_h(x)$ be the numerical solution of (15) at $t=t_{\max }$ obtained using the DG spatial discretization with $p = 2$ in (11) and solving the resulting semidiscretized ODE system using one of the standard RK methods as mentioned above. The final time $t_{\max } = 0.1$ is chosen such that the exact solution is still a smooth function (before the onset of shock). Therefore no regularization or limiting is expected to be essential to witness high order convergence. The exact solution at $t_{\max }$ shown in Fig. 2a is obtained by the method of characteristics together with a Newton method. Thus one would expect the error

15c $$\begin{aligned} {e_h} := \Vert u(\cdot ,0.1)-u_h\Vert _{L^2([0,1])} \end{aligned}$$

to go to zero at a rate of ${\mathcal {O}}(h^3)$. However our observations in Fig. 2b run counter to that expectation. Figure 2b reports the rates we observed when two standard time stepping schemes were used to solve (14), namely the two-stage (RK2) and the four-stage (RK4) explicit Runge–Kutta time stepping schemes. Although we see third order convergence for the first few refinement steps, the rate eventually drops to first order for both methods. We shall return to this example in Sect. 7.1 after developing a method without this convergence reduction.

## Structure aware Runge–Kutta type methods

In this section, we develop specialized Runge–Kutta type schemes that do not show the above mentioned order loss of classical Runge–Kutta schemes.

We motivate the definition of the new scheme by reformulating (12) in terms of two variables $Z({\hat{t}})$ and $Y({\hat{t}})$, defined by

$$\begin{aligned} Z({\hat{t}}) = M_0(U({\hat{t}})), \qquad Y({\hat{t}}) = M({\hat{t}}, U({\hat{t}})) = Z({\hat{t}}) - \hat{t}M_1(U(\hat{t})). \end{aligned}$$

Then (12) implies

16 $$\begin{aligned} Y' = A(U({\hat{t}})), \qquad Z' = A(U({\hat{t}})) + ({\hat{t}} M_1(U({\hat{t}})))', \end{aligned}$$

together with the initial conditions $Y(0) = Z(0) = M_0(U_0).$ Here and throughout we use primes ($'$) to abbreviate $d / d{\hat{t}}$. The key idea is to avoid the inversion of the time-dependent *M* at all ${\hat{t}}$, limiting the inversion to just that of $M_0$. Assuming we can compute the time-independent inverse $M_0^{-1}$, we define

$$\begin{aligned} {\tilde{A}}= A \circ M_0^{-1}, \qquad {\tilde{M}}_1 = M_1 \circ M_0^{-1}. \end{aligned}$$

Then, (16) yields the following ODE system for *Y* and *Z* on $0< {\hat{t}} < 1$:

17a $$\begin{aligned} Z'&= {\tilde{A}}( Z({\hat{t}})) + ({\hat{t}} {\tilde{M}}_1(Z({\hat{t}})))',&Z(0) = Y_0,\end{aligned}$$

17b $$\begin{aligned} Y'&= {\tilde{A}}( Z({\hat{t}})),&Y(0) = Y_0, \end{aligned}$$

where $$Y_0 = M_0(U_0)$$.

Integrating the equations of (17) from 0 to $\tau$, we obtain

18a $$\begin{aligned} Z(\tau )&= Z(0) + \tau {\tilde{M}}_1(Z(\tau )) + \int _0^{\tau } {\tilde{A}}(Z(s)) \,\mathrm {d}s,\end{aligned}$$

18b $$\begin{aligned} Y(\tau )&= Y(0)+ \int _0^{\tau } {\tilde{A}}(Z(s))\, \mathrm {d}s. \end{aligned}$$

The new scheme, defined below, may be thought of as motivated by quadrature approximations to the integrals above. Note that we are only interested in such quadratures that result in explicit schemes. Moreover, we must also approximate $\tau {\tilde{M}}_1(Z(\tau ))$ by an extrapolation formula that uses prior values of *Z*,  in order to keep the scheme explicit.

### Definition 1

Given an initial condition $Y_0$, *an s-stage SARK method* for (17) computes

19a $$\begin{aligned} Z_i&= Y_0 + \tau \sum _{j<i} d_{ij}{\tilde{M}}_1(Z_j) + \tau \sum _{j<i}a_{ij}{\tilde{A}}(Z_j), \qquad 1\le i \le s, \end{aligned}$$

19b $$\begin{aligned} Y_\tau&= Y_0 + \tau \sum _{i=1}^s b_i {\tilde{A}}(Z_i). \end{aligned}$$

This explicit method is determined by the coefficient matrices $b \in {\mathbb {R}}^{s \times 1}$, $\mathcal {A} \in {\mathbb {R}}^{s \times s}$, and $\mathcal {D} \in {\mathbb {R}}^{s \times s}$: $$\begin{aligned} b = (b_1,\dots ,b_s), \quad \mathcal {A} = \begin{pmatrix} 0 &{} &{} &{} \\ a_{21} &{} 0 &{} &{}\\ \vdots &{} \ddots &{} 0 &{} \\ a_{s1} &{} \dots &{} a_{s,s-1} &{} 0\\ \end{pmatrix}, \quad \mathcal {D} = \begin{pmatrix} 0 &{} &{} &{} \\ d_{21} &{} 0 &{} &{}\\ \vdots &{} \ddots &{} 0 &{} \\ d_{s1} &{} \dots &{} d_{s,s-1} &{} 0\\ \end{pmatrix}. \end{aligned}$$

Hence we use  instead of the standard Butcher tableau  to express our scheme. Here we restrict ourselves to schemes where $c \in {\mathbb {R}}^s$ is set by the consistency condition

$$\begin{aligned} c_i= \sum _{j=1}^{i-1} a_{ij}. \end{aligned}$$

In the next section, we shall develop a theory to choose appropriate values of $a_{ij}, d_{ij},$ and $b_i$. There, Sect. 5.4 contains some specific examples of SARK scheme tableaus.

## Order conditions for the scheme

Appropriate values of $a_{ij}, d_{ij},$ and $b_i$ can be found by order conditions obtained by matching terms in the Taylor expansions of the exact solution $Y(\tau )$ and the discrete solution $Y_\tau$. To derive these order conditions we follow the general methodology laid out in Ref. [9]. For this, we need to first compute the derivatives of the exact flow (in Sect. 5.1), then the derivatives of the discrete flow (in Sect. 5.2), followed by the formulation of resulting order conditions (in Sect. 5.3).

### Derivatives of the exact solution

Continuing to use primes ($'$) for total derivatives with respect to a single variable like $d/d \tau$, to ease the tedious calculations below, we shall also employ the *n*th order Frechet derivative of a function $g: D \subset {\mathbb {R}}^m \rightarrow V$, for some vector space *V*. It is denoted by $g^{(n)}(z): {\mathbb {R}}^m \times \cdots \times {\mathbb {R}}^m \rightarrow V$ and defined by the symmetric multilinear form

$$\begin{aligned} g^{(n)}(z)(v_1, \ldots , v_n) = \sum _{i_1, i_2, \ldots , i_n=1}^m \frac{ \partial ^n g(z)}{ \partial x_{i_1} \cdots \partial x_{i_n}} [v_1]_{i_1} \ldots [v_n]_{i_n} \end{aligned}$$

for any $v_1, \ldots , v_n \in {\mathbb {R}}^m.$ Whenever *g* and $z: (0,1) \rightarrow {\mathbb {R}}^m$ are sufficiently smooth for the derivatives below to exist continuously, we have the following formulae.

20a $$\begin{aligned} \frac{d}{d \tau } g(z(\tau ))&= g^{(1)}(z(\tau ))(z'(\tau )),\end{aligned}$$

20b $$\begin{aligned} \frac{d^2}{d \tau ^2} g(z(\tau ))&= g^{(2)}(z(\tau ))(z'(\tau ), z'(\tau )) + g^{(1)}(z(\tau )(z''(\tau )), \end{aligned}$$

20c $$\begin{aligned} \frac{d^3}{d \tau ^3} g(z(\tau ))&= g^{(3)}(z(\tau ))(z'(\tau ), z'(\tau ), z'(\tau )) \nonumber \\& \quad + 3 g^{(2)}(z(\tau ))(z'(\tau ), z''(\tau )) + g^{(1)}(z(\tau ))(z'''(\tau )),\end{aligned}$$

20d $$\begin{aligned} \frac{d^4}{d \tau ^4} g(z(\tau )) &= g^{(4)}(z(\tau ))(z'(\tau ), z'(\tau ), z'(\tau ), z'(\tau )) \nonumber \\& \quad + 6 g^{(3)}(z(\tau ))(z'(\tau ), z'(\tau ), z''(\tau )) + 4 g^{(2)}(z(\tau ))(z'(\tau ), z'''(\tau )) \nonumber \\& \quad + 3 g^{(2)}(z(\tau )) (z''(\tau ), z''(\tau )) + g^{(1)}(z(\tau ))(z''''(\tau )). \end{aligned}$$

These formulae can be derived by repeated application of the chain rule (or by applying the Faá di Bruno formula). We will also need to use

21 $$\begin{aligned} \frac{d^k}{d \tau ^k}\big ( \tau g(z(\tau )) \big ) = \tau \frac{d^k}{d \tau ^k} g(z(\tau )) + k \frac{d^{k-1}}{d \tau ^{k-1}} g(z(\tau )), \end{aligned}$$

which is a simple consequence of the Leibniz rule.

We start by computing the derivatives of $Z(\tau )$ at $\tau =0$. To express such derivatives concisely, we introduce the notation

$$\begin{aligned} \alpha = {\tilde{A}}(Z(0)), \qquad \alpha ^{{(n)}} (v_1, \ldots , v_n) = {\tilde{A}}^{(n)}(Z(0))(v_1, \ldots , v_n),\\ \mu = {\tilde{M}}_1(Z(0)), \qquad \mu ^{{(n)}} (v_1, \ldots , v_n) = {\tilde{M}}^{(n)}(Z(0))(v_1, \ldots , v_n). \end{aligned}$$

From (17a), it is immediate that $Z'(0) = {\tilde{A}}(Z(0)) + {\tilde{M}}(Z(0))$. Thus,

22a $$\begin{aligned} Z'(0)&= \alpha + \mu . \end{aligned}$$

For the next derivative, we differentiate (17a) twice to get $Z''(\tau ) = ({\tilde{A}}(Z(\tau ))' + (\tau {\tilde{M}}_1(Z(\tau )))''.$ Calculating the latter using (21), simplifying using (20), and evaluating at $\tau =0$, we obtain

22b $$\begin{aligned} Z''(0) = (\alpha ^{{(1)}} + 2 \mu ^{{(1)}})(\alpha + \mu ). \end{aligned}$$

By the same procedure, starting with $Z'''(\tau ) = ({\tilde{A}}(Z(\tau ))'' + (\tau {\tilde{M}}_1(Z(\tau )))'''$ and using (21) and (20), we also have

22c $$\begin{aligned} Z'''(0) = (\alpha ^{{(2)}} + 3\mu ^{{(2)}})(\alpha + \mu , \alpha + \mu ) + (\alpha ^{{(1)}} + 3 \mu ^{{(1)}}) \big ( (\alpha ^{{(1)}} + 2 \mu ^{{(1)}} )(\alpha + \mu ) \big ). \end{aligned}$$

Armed with (22), we proceed to compute the derivatives of $Y$. Obviously, (17b) implies

23a $$\begin{aligned} Y'(0) = {\tilde{A}}(Z(0)) = \alpha . \end{aligned}$$

Differentiating (17b) again, using (20), and evaluating at $\tau =0$ using the previously computed derivatives of *Z* in (22), we also get

23b $$\begin{aligned} Y''(0)&= \alpha ^{{(1)}} (\alpha + \mu ) \end{aligned}$$

23c $$\begin{aligned} Y'''(0)&= \alpha ^{{(2)}} (\alpha +\mu , \alpha +\mu ) + \alpha ^{{(1)}} \big ( (\alpha ^{{(1)}} + 2 \mu ^{{(1)}} )(\alpha + \mu ) \big ). \end{aligned}$$

### Derivatives of the discrete flow

The next task is to compute the coefficients of the Taylor expansion of the function $Y_\tau$ defined in (19b). The arguments $Z_i$ in (19b) are also functions of $\tau$, as given by (19a). Therefore, in what follows, we first differentiate $Z_i \equiv Z_i(\tau )$ and then $Y_\tau$.

Obviously, $Z_i(0)$ and *Z*(0) coincide, so we will focus on the first and higher derivatives of $Z_i$ at $\tau =0$. To this end, we differentiate (19a) *k* times to get

$$\begin{aligned} \frac{d^k Z_i}{d \tau ^k} = \sum _{j<i} \bigg [ d_{ij}\frac{d^k}{ d\tau ^k} (\tau {\tilde{M}}_1(Z_j(\tau )))+ a_{ij} \frac{d^k}{ d \tau ^k}(\tau {\tilde{A}}(Z_j(\tau )))\bigg ]. \end{aligned}$$

Using (21) for $k=1,2, 3$, then (20), and evaluating at $\tau =0$ we obtain

24a $$\begin{aligned} Z_i'(0)&= \sum _{j < i} d_{ij} \mu + a_{ij} \alpha \end{aligned}$$

24b $$\begin{aligned} Z_i''(0)&= 2 \sum _{j< i} \sum _{k < j} \big ( d_{ij}\mu ^{{(1)}} + a_{ij}\alpha ^{{(1)}} \big )(d_{jk} \mu + a_{jk} \alpha )\end{aligned}$$

24c $$\begin{aligned} Z_i'''(0)&= 3 \sum _{j< i} \sum _{k< j} \sum _{l< j} \big ( d_{ij} \mu ^{{(2)}} + a_{ij} \alpha ^{{(2)}} \big ) ( d_{jk} \mu + a_{jk} \alpha , d_{jl} \mu + a_{jl} \alpha )\nonumber \\&+ 6 \sum _{j< i} \sum _{k< j} \sum _{l < k} \big ( d_{ij} \mu ^{{(1)}} + a_{ij} \alpha ^{{(1)}} \big ) \big ( (d_{jk} \mu ^{{(1)}} + a_{jk} \alpha ^{{(1)}}) (d_{kl} \mu + a_{lk}\alpha ) \big ). \end{aligned}$$

Next, we focus on $Y_\tau$. By (19b),

$$\begin{aligned} \frac{d^k Y_\tau }{d \tau ^k} = \sum _{i=1}^s b_i \frac{d^k }{d \tau ^k}({\tilde{A}}(Z_i(\tau ))). \end{aligned}$$

Using (20), and evaluating the resulting terms at $\tau = 0$ by means of (24), we obtain

25a $$\begin{aligned} Y_\tau '(0)&= \sum _{i=1}^s b_i \alpha ,\end{aligned}$$

25b $$\begin{aligned} Y_\tau ''(0)&= 2\sum _{i=1}^s \sum _{j < i} b_i\alpha ^{{(1)}}(d_{ij} \mu + a_{ij} \alpha ),\end{aligned}$$

25c $$\begin{aligned} Y_\tau '''(0)&= 3 \sum _{i=1}^s\sum _{j< i} \sum _{k< i} b_i \alpha ^{{(2)}}(d_{ij} \mu + a_{ij} \alpha ,d_{ik} \mu + a_{ik} \alpha ) \nonumber \\&\quad + 6 \sum _{i=1}^s\sum _{j< i} \sum _{k < j} b_i\alpha ^{{(1)}} \big ( (d_{ij}\mu ^{{(1)}} + a_{ij} \alpha ^{{(1)}} ) (d_{jk} \mu + a_{jk} \alpha )\big ). \end{aligned}$$

### Formulation of order conditions

To obtain a specific method, we find values for $a_{ij}, d_{ij}$ and $b_i$ by matching the coefficients in the Taylor expansions of $Y(\tau )$ and $Y_\tau$. Note that $Y_\tau (0) = Y_0 = Y(0)$, so the 0th order coefficients match.

The next terms in the Taylor expansions will match if $Y'(0)=Y_\tau '(0)$. For this it is sufficient that

26 $$\begin{aligned} \sum _{i=1}^s b_i = 1. \end{aligned}$$

because of (23a) and (25a). To match the third terms in the Taylor expansions, equating (23b) and (25b),

$$\begin{aligned} \alpha ^{{(1)}} (\alpha ) + \alpha ^{{(1)}} (\mu ) = \sum _{i=1}^s \sum _{j < i} 2b_i d_{ij} \alpha ^{{(1)}} (\mu ) + 2 b_ia_{ij} \alpha ^{{(1)}} (\alpha ). \end{aligned}$$

Equating the coefficients of $\alpha ^{{(1)}} (\alpha )$ and $\alpha ^{{(1)}} (\mu )$, we conclude that $Y''(0) = Y_\tau ''(0)$ if

27 $$\begin{aligned} 2\sum _{i=1}^s \sum _{j<i} b_i d_{ij} = 1 \quad \text {and} \quad 2\sum _{i=1}^s\sum _{j<i} b_ia_{ij} = 1. \end{aligned}$$

If one desires to further match the next higher order terms, $Y_\tau '''(0) = Y'''(0)$, then the expressions in (23c) and (25c) must be equated, i.e.,

$$\begin{aligned}& \alpha ^{{(2)}} (\alpha ,\! \alpha ) + 2 \alpha ^{{(2)}} (\alpha ,\!\mu ) + \alpha ^{{(2)}} (\mu ,\! \mu )\\& \qquad + \alpha ^{{(1)}} (\alpha ^{{(1)}} (\alpha )) + \alpha ^{{(1)}} (\alpha ^{{(1)}} (\mu )) + 2 \alpha ^{{(1)}} (\mu ^{{(1)}} (\alpha ) ) + 2 \alpha ^{{(1)}} (\mu ^{{(1)}} (\mu )) \\& \quad = \sum _{i=1}^s \sum _{j< i} \sum _{k< i} \bigg [ 3 b_i d_{ij} d_{ik} \alpha ^{{(2)}} (\mu ,\! \mu ) + 6 b_i d_{ij} a_{ik} \alpha ^{{(2)}} (\mu , \alpha ) + 3 b_i a_{ij} a_{ik} \alpha ^{{(2)}} (\alpha , \alpha ) \bigg ] \\& \quad + 6 \sum _{i=1}^s \sum _{j< i} \sum _{k < j} \bigg [ b_i d_{ij}d_{jk} \alpha ^{{(1)}} (\mu ^{{(1)}} (\mu )) + b_i d_{ij}a_{jk} \alpha ^{{(1)}} (\mu ^{{(1)}} (\alpha )) \\& \qquad + b_i a_{ij}d_{jk} \alpha ^{{(1)}} (\alpha ^{{(1)}} (\mu )) + b_i a_{ij}a_{jk} \alpha ^{{(1)}} (\alpha ^{{(1)}} (\alpha )) \bigg ]. \end{aligned}$$

For this equality to hold, the following seven conditions are sufficient as can be seen by equating the coefficients of $$\alpha ^{{(2)}} (\alpha , \alpha ),$$$$\alpha ^{{(2)}} (\mu , \mu ),$$$$\alpha ^{{(2)}}(\alpha , \mu ),$$$$\alpha ^{{(1)}} (\alpha ^{{(1)}} (\alpha )),$$$$\alpha ^{{(1)}} (\alpha ^{{(1)}} (\mu )),$$$$\alpha ^{{(1)}} (\mu ^{{(1)}} (\alpha ) ),$$ and $$\alpha ^{{(1)}} (\mu ^{{(1)}} (\mu ))$$, respectively:

28a $$\begin{aligned} 3 \sum _{i=1}^s b_i \Big (\sum _{j<i} a_{ij}\Big )^2 = 1, \end{aligned}$$

28b $$\begin{aligned} 3 \sum _{i=1}^s b_i \Big (\sum _{j<i} d_{ij}\Big )^2 = 1, \end{aligned}$$

28c $$\begin{aligned} 3 \sum _{i=1}^s b_i \Big (\sum _{j<i} a_{ij}\Big )\Big (\sum _{j<i} d_{ij}\Big ) = 1,\end{aligned}$$

28d $$\begin{aligned} 6 \sum _{i=1}^s \sum _{j<i}\sum _{k<j} b_ia_{ij}a_{jk} = 1,\end{aligned}$$

28e $$\begin{aligned} 6 \sum _{i=1}^s \sum _{j<i}\sum _{k<j} b_ia_{ij}d_{jk} = 1,\end{aligned}$$

28f $$\begin{aligned} 3 \sum _{i=1}^s \sum _{j<i}\sum _{k<j} b_id_{ij}a_{jk} = 1,\end{aligned}$$

28g $$\begin{aligned} 3 \sum _{i=1}^s \sum _{j<i}\sum _{k<j} b_id_{ij}d_{jk} = 1. \end{aligned}$$

Thus, we have proved the following result, which summarizes our discussions on order conditions.

#### Theorem 1

Whenever ${\tilde{A}}$ and ${\tilde{M}}$ are smooth enough for the derivatives below to exist continuously,

1. the condition (26) implies $Y'(0) = Y_\tau '(0),$
2. the conditions of (27) imply $Y''(0) = Y_\tau ''(0),$ and
3. the conditions of (28) imply $Y'''(0) = Y_\tau '''(0)$.

### Examples of methods up to third order

Observe that the standard order conditions of Runge–Kutta methods are a subset of the order conditions derived in Sect. 5.3. Thus we base our SARK methods on existing Runge–Kutta methods. Below, we shall refer to an *s*-stage SARK method based on an existing Runge–Kutta method called “RKname” as “SARK(*s*, RKname)”.

A second order two-stage SARK method can be derived from a second order Runge–Kutta method once we find $d_{ij}$ satisfying the additional condition

29 $$\begin{aligned} 2\sum _{i=1}^2 \sum _{j<i} b_i d_{ij} = 1 \quad \Leftrightarrow \quad b_2 d_{21} = \frac{1}{2}, \end{aligned}$$

which was introduced in (27). For example, one may start with the standard explicit midpoint rule and select $d_{21}=1/2$ to satisfy (29), thus arriving at the “SARK(2, midpoint)” method, listed first in Table 1. The table continues on to display further such methods obtained from other well-known second order Runge–Kutta schemes.

**Table 1:**
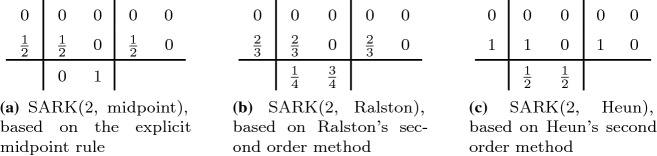
Two-stage SARK methods

**Table 2:**
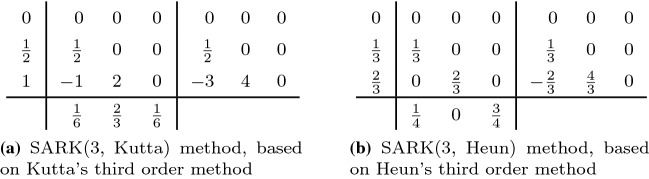
Three-stage SARK methods

The third order SARK methods in Table 2 are based on known third order Runge–Kutta methods with three stages. The additional coefficients $d_{ij}$ are chosen, such that (27)–(28) are satisfied.

### Application of multiple steps within a tent

Recall that the ODE system we need to solve within one mapped tent is (17) for $0< {\hat{t}} < 1.$ Since the ${\hat{t}}$ interval is not small, we subdivide it into *r* subintervals and use the previously described *s*-stage SARK scheme within each subinterval, as described next.

We subdivide the unit interval [0, 1] into *r* subintervals

$$\begin{aligned}{}[{\hat{t}}_k, {\hat{t}}_{k+1}], \quad k=0,1, \ldots , r-1, \quad \text { where } {\hat{t}}_k = \frac{k}{r}, \end{aligned}$$

and apply (1) within each subinterval as described next.

**Fig. 3:**
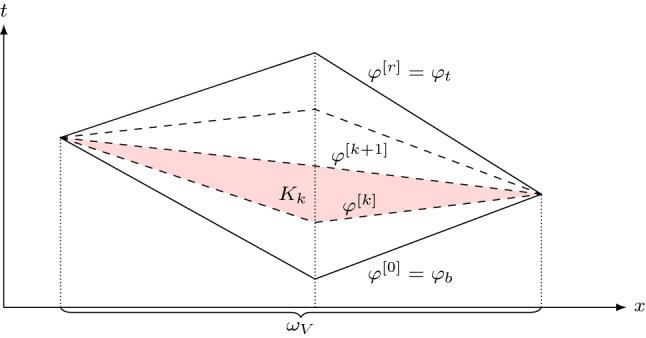
Illustration of the subtent $K_k$ (shaded) defined in (30). It is the image under $\varPhi$ of the tensor product domain ${\hat{K}}_k = {{\omega _V}}\times (\hat{t}_k,\hat{t}_{k+1})$.

First observe that the above splitting of the unit ${\hat{t}}$-interval corresponds to subdividing the original tent *K*, as given by (4), into *r* “subtents” (see Fig. 3) of the form

30 $$\begin{aligned} K_k = \{ (x, t): \; x \in {{\omega _V}}, \;\varphi ^{[k]} \le t \le \varphi ^{[k+1]}\} \end{aligned}$$

where $\varphi ^{[k]} = \varphi ({\hat{t}}_k)$. Clearly $\varphi ^{[0]} = \varphi _b$ and $\varphi ^{[r]} = \varphi _t$.

We then apply (1) to each of these subtents. Accordingly, let $M_{0, [k]}$ be defined by (11a) after replacing $\varphi _b$ by $\varphi ^{[k]}$. Keeping the same definition of *A* and $M_1$, let ${\tilde{M}}_1^{[k]} = M_1 \circ M_{0, [k]}^{-1}$, ${\tilde{A}}^{[k]} = A \circ M_{0, [k]}^{-1}$, and $\tau ^{[k]} = {\hat{t}}_{k+1} - {\hat{t}}_k$. Then the application of (1) on each interval $[{\hat{t}}_k, {\hat{t}}_{k+1}]$ results in the following algorithm.

#### Algorithm 1

1. If the input is $Y_0$, an approximation to *Y*(0) at the tent bottom, then set $Y^{[0]} = Y_0$. If the input is $U_0$, an approximation to *U*(0) at the tent bottom, then set $Y^{[0]} = Y_0 = M_0(U_0)$.
2. For $$k = 0, 1, \ldots , r-1$$ do:
  - For $$i = 1, 2, \ldots , s$$, compute
    $$\begin{aligned} Z_i^{[k]} = Y^{[k]} + \tau ^{[k]} \sum _{j=1}^{i-1} d_{ij}{\tilde{M}}_1^{[k]}(Z_j^{[k]}) + \tau ^{[k]} \sum _{j=1}^{i-1} a_{ij}{\tilde{A}}^{[k]}(Z_j^{[k]}). \end{aligned}$$
  - Compute
    $$\begin{aligned} Y^{[k+1]} = Y^{[k]} + \tau ^{[k]} \sum _{i=1}^s b_i {\tilde{A}}^{[k]}(Z_i^{[k]}). \end{aligned}$$
3. Set
  $$\begin{aligned} Y_1^{r, s} = Y^{[r]}. \end{aligned}$$
  Output this as the approximation to *Y*(1) at the tent top.

We conclude this section by defining the propagation operators of the above algorithm, which we shall use later. At step *k*, we define the (generally nonlinear) partial propagation operator $T^{[k+1]} : V_h({\omega _V}) \rightarrow V_h({\omega _V})$, using the intermediate quantities in the algorithm:

31a $$\begin{aligned} T^{[k+1]} (Y^{[k]}) = Y^{[k+1]}. \end{aligned}$$

Let the total propagation operator on the tent $T : V_h({\omega _V}) \rightarrow V_h({\omega _V})$ be defined by

31b $$\begin{aligned} T = T^{[r]} \circ \cdots \circ T^{[2]} \circ T^{[1]}. \end{aligned}$$

Clearly, the input and output of the algorithm are related to *T* by

32 $$\begin{aligned} Y_1^{r, s} = T(Y_0). \end{aligned}$$

## Investigation of discrete stability

This section is devoted to remarks on the stability of the new SARK schemes. While it is common to study stability of ODE solvers by applying them to a simple scalar ODE, keeping our application of spatially varying hyperbolic solutions in mind, we consider changes in an energy-like measure on the solution $U({\hat{t}})$. Recall that $U(\hat{t})\in {\mathbb {R}}^m$ is the coefficient vector of the basis expansion of the mapped finite element solution ${\hat{u}}(x,\hat{t})\in V_h({{\omega _V}})$, as defined by (10). We limit ourselves to the case where the energy-like quantity

33 $$\begin{aligned} \Vert U(\hat{t})\Vert _{M(\hat{t})}^2:= \int _{{\omega _V}}M({\hat{t}}, U) \cdot {\hat{u}} = \int _{{\omega _V}}\left( M_0 (U) - \hat{t}M_1(U)\right) \cdot {\hat{u}} \end{aligned}$$

is a *norm* and (the generally nonlinear operators) $M, M_0$ and $M_1$ defined in (13), (11a) and (11b), respectively, are linear, so that we may rewrite $M({\hat{t}}, U) = M({\hat{t}}) U$ using the linear operator $M({\hat{t}}):= M_0 - {\hat{t}} M_1 : {\mathbb {R}}^m \rightarrow V_h({\omega _V})$. For many standard linear hyperbolic systems, the causality condition—see (2)—can be used to easily show that $M({\hat{t}})$ is identifiable with a symmetric positive definite matrix so that (33) indeed defines a norm. In the special case of $g(v) = v$, we note that on flat advancing fronts, where $\varphi (x,\hat{t})$ is independent of *x* for some fixed $\hat{t}$, (33) reduces to

$$\begin{aligned} \Vert U({\hat{t}})\Vert _{M(\hat{t})}^2=\int _{{\omega _V}}{\hat{u}} \cdot {\hat{u}}, \end{aligned}$$

so $\Vert U({\hat{t}}) \Vert _{M(\hat{t})}$ becomes the familiar spatial $L^2$ norm of $\hat{u}(\cdot , {\hat{t}})$.

### Our procedure to study linear stability

Stability of the scheme within a tent can be understood by studying the discrete analogue of the ratio $\Vert U(1)\Vert _{M(1)} / \Vert U(0)\Vert _{M(0)}$ for all possible initial data *U*(0). This amounts to studying the norm of the discrete propagation operator for *U*, which we proceed to formulate. First, recall the connection between *U* and *Y*, namely $Y({\hat{t}}) = M({\hat{t}}) U({\hat{t}})$. Algorithm 1, takes as input an approximation $U_0$ to *U*(0) at the tent bottom and outputs $Y_1^{r, s}$, an approximation to *Y*(1) at the tent top. Hence the associated approximation to *U*(1) is

$$\begin{aligned} U^{r,s}_1 := M(1)^{-1} Y_1^{r, s}. \end{aligned}$$

Next, recall the discrete propagation operator defined by (31). It is now a linear operator that maps $Y_0= M(0) U_0$ to $Y_1^{r, s}$ according to (32). Define the *tent propagation matrix*$S : {\mathbb {R}}^m \rightarrow {\mathbb {R}}^m$ by

34 $$\begin{aligned} S = M(1)^{-1} T M(0). \end{aligned}$$

Clearly, (32) implies that

35 $$\begin{aligned} U_1^{r,s} = S \,U_0. \end{aligned}$$

The discrete analogue of $\Vert U(1)\Vert _{M(1)} / \Vert U(0)\Vert _{M(0)}$ is $\Vert U_1^{r, s}\Vert _{M(1)} / \Vert U_0\Vert _{M(0)}$ which can be bounded using the following norm of *S*:

$$\begin{aligned} \Vert S \Vert _{L(M(0), M(1))} \,= \sup _{0 \ne W \in {\mathbb {R}}^m} \frac{ \Vert S W \Vert _{M(1)}}{ \Vert W \Vert _{M(0)}}. \end{aligned}$$

It is immediate from (35) that $\Vert U_1^{r,s}\Vert _{M(1)} \le \Vert S \Vert _{L(M(0), M(1))} \Vert U_0\Vert _{M(0)}$. Thus the study of stability of SARK schemes is reduced to computing estimates for the norm of *S*.

We now describe how we computed the norm of *S* for some examples below. Writing ${\hat{v}} (x, \hat{t}) = \sum _{i=1}^m V_i(\hat{t}) \psi _i(x)$ and ${\hat{w}} (x, \hat{t}) = \sum _{i=1}^m W_i(\hat{t}) \psi _i(x)$, in analogy with the basis expansion of ${\hat{u}}$ in (10), let ${\mathbb {M}}_{{\hat{t}}}$ be the $m\times m$ symmetric positive definite matrix satisfying $W^\top {\mathbb {M}}_{{\hat{t}}} V = \int _{\omega _V}M({\hat{t}}) V \cdot {\hat{w}}$. Then

$$\begin{aligned} \Vert S \Vert ^2_{L(M(0), M(1))}&= \sup _{0 \ne W \in {\mathbb {R}}^m} \frac{ (S W)^\top {\mathbb {M}}_1 (SW)}{ W^\top {\mathbb {M}}_0 W} \\&= \sup _{0 \ne W \in {\mathbb {R}}^m} \frac{ W^\top (S^\top {\mathbb {M}}_1 S) W}{ W^\top {\mathbb {M}}_0 W} \\&= \sup \{ |\lambda |: \;\exists 0 \ne X \in {\mathbb {R}}^m \text { satisfying } (S^\top {\mathbb {M}}_1 S) X = \lambda {\mathbb {M}}_0 X\}. \end{aligned}$$

Thus, to investigate the stability of a scheme, we computed $T^{[k]}$ from the scheme’s Butcher-like tableau, then *T* by (31b), followed by *S* per (34), and finally, the square root of the spectral radius of ${\mathbb {M}}_0^{-1} (S^\top {\mathbb {M}}_1 S)$, which equals $\Vert S \Vert _{L(M(0), M(1))}$ as shown above. We expand on the first of these steps in the next few subsections by displaying $T^{[k]}$ for some SARK schemes and end this section by reporting our numerical estimates for $\Vert S \Vert _{L(M(0), M(1))}$ for an example.

### Propagation operator of two-stage SARK methods

For an arbitrary two-stage SARK method the only non-zero coefficients are $b_1, b_2, a_{21}, d_{21}$. For a given $Y^{[k]}=M_{0,[k]}U^{[k]}$ we obtain

$$\begin{aligned} Z_1^{[k]}&= Y^{[k]}, \\ Z_2^{[k]}&= Y^{[k]} + \tau ^{[k]} d_{21}{\tilde{M}}_1^{[k]} Z_1^{[k]} + \tau ^{[k]} a_{21}{\tilde{A}}^{[k]} Z_1^{[k]}\\&= \left( I + \tau ^{[k]} \left( d_{21}{\tilde{M}}_1^{[k]} + a_{21}{\tilde{A}}^{[k]}\right) \right) Y^{[k]}, \end{aligned}$$

with the identity matrix $I\in {\mathbb {R}}^{m\times m}$. The propagation from $\hat{t}_k$ to $\hat{t}_{k+1}$ reads

$$\begin{aligned} Y^{[k+1]}&= Y^{[k]} + \tau ^{[k]} \left( b_1 {\tilde{A}}^{[k]} Z_1^{[k]} + b_2 {\tilde{A}}^{[k]} Z_2^{[k]}\right) \\&= \left( I + \tau ^{[k]}(b_1+b_2) {\tilde{A}}^{[k]} + (\tau ^{[k]})^2 {\tilde{A}}^{[k]}\left( b_2d_{21}{\tilde{M}}_1^{[k]} + b_2a_{21} {\tilde{A}}^{[k]}\right) \right) Y^{[k]} \\&= \left( I + \tau ^{[k]}{\tilde{A}}^{[k]} + \tfrac{1}{2}(\tau ^{[k]})^2 {\tilde{A}}^{[k]}\left( {\tilde{M}}_1^{[k]} + {\tilde{A}}^{[k]} \right) \right) Y^{[k]}, \end{aligned}$$

where we used the order conditions (26) and (27) for second order methods. This results in the propagation matrix

$$\begin{aligned} T^{[k]}&= I + \tau ^{[k]}{\tilde{A}}^{[k]} + \tfrac{1}{2}(\tau ^{[k]})^2 {\tilde{A}}^{[k]}\left( {\tilde{M}}_1^{[k]} + {\tilde{A}}^{[k]} \right) , \end{aligned}$$

such that $$Y^{[k+1]} = T^{[k]}Y^{[k]}$$.

### Propagation operator of three-stage SARK methods

A similar calculation for three-stage SARK methods, using the order conditions (26)–(28), leads to the propagation matrix

$$\begin{aligned} T^{[k]} =I&+ \tau ^{[k]}{\tilde{A}}^{[k]} + \tfrac{1}{2}(\tau ^{[k]})^2 {\tilde{A}}^{[k]}\left( {\tilde{M}}_1^{[k]} + {\tilde{A}}^{[k]} \right) \\&+ \tfrac{1}{6}(\tau ^{[k]})^3{\tilde{A}}^{[k]}\left( 2{\tilde{M}}_1^{[k]} + {\tilde{A}}^{[k]} \right) \left( {\tilde{M}}_1^{[k]} + {\tilde{A}}^{[k]} \right) . \end{aligned}$$

### Discrete stability measure for a model problem

We report the practically observed values of the previously described stability measure (namely the norm $\Vert S \Vert _{L(M(0), M(1))}$) for some SARK schemes applied to the two-dimensional convection equation

$$\begin{aligned} \partial _t u(x,t) + {\mathrm {div}}_x\left( b\,u(x,t)\right) = 0,\quad \forall (x,t)\in \Omega _0\times (0,t_{\max }], \end{aligned}$$

with $\Omega _0=[0,1]^2, t_{\max }=0.05$, the flux field $b=(1,1)^\top$ and periodic boundary conditions. The time slab $\Omega = \Omega _0\times (0,t_{\max })$ is filled with tents. Within each such tent $K_i$, let $C_i$ denote the norm $\Vert S \Vert _{L(M(0), M(1))}$ computed with *S*, *M*(0),  and *M*(1) specific to that tent. We expect $C_i$ to be close to one for a stable method. Let

36 $$\begin{aligned} {\bar{C}} := \max _{i} \; \left\{ C_i - 1 \right\} , \end{aligned}$$

where the maximum is taken over all tents in the time slab. To gain an understanding of practical stability, we examine the values of ${\bar{C}}$ as a function of the number of SARK stages (*s*), polynomial degree (*p*), and more importantly, the number of substeps per tent (*r*).

**Fig. 4:**
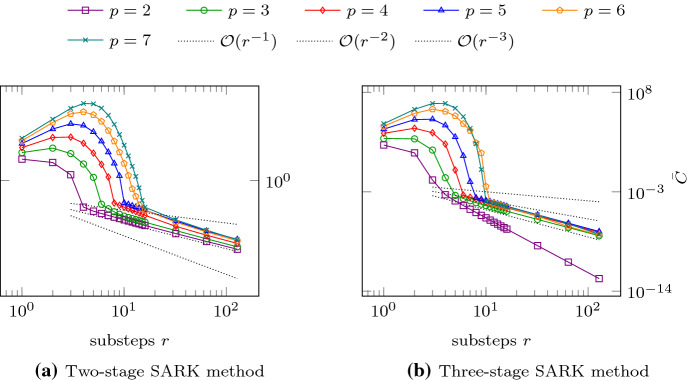
Observed dependence of ${\bar{C}}$ on *r* for $p=2,3,4,5,6,7$ and $s=2, 3.$

In all our numerical experiments, we observed that on each tent, for a fixed *s*, the norm $\Vert S \Vert _{L(M(0), M(1))}$ tends to 1 with increasing number of substeps *r*, and moreover, we discovered a dependence of the following form

$$\begin{aligned} \Vert S \Vert _{L(M(0), M(1))} = 1 + \mathcal {O}\left( r^{-s}\right) \end{aligned}$$

on each tent $K_i$. Therefore, we organize our report on numerical stability observations into plots of values of ${\bar{C}}$ as a function of *r*. We do so for two SARK methods, one with $s=2$ and another with $s=3$. The results are displayed in Fig. 4. After a prominent preasymptotic region, we observe that ${\bar{C}}$, as a function of *r*, exhibits the rate $\mathcal {O}\left( r^{-s}\right)$ in all cases, except one.

The exceptional case is the case $p=2$ in Fig. 4b, where the stability measure approaches the ideal value of 1 much faster. We do not have an explanation for this observation.

Note that all the plotted curves in Fig. 4 shift to the top and right as *p* increases, i.e., the number of substeps *r* required to keep the same stability measure ${\bar{C}}$ increases with *p*. This behavior is akin to the *p*-dependence of the CFL-conditions of standard time stepping schemes.

## Numerical results

In this section, we collect our observations on the performance of the new SARK schemes, on the one-dimensional Burgers’ equation (in Sect. 7.1) and the two-dimensional Euler system (in Sects. 7.2–7.3). While Sect. 7.2 focuses on the study of convergence rates for a smooth Euler solution, Sect. 7.3 presents the application of SARK scheme on the computationally challenging problem of simulating a Mach 3 wind tunnel with a forward-facing step.

### Convergence rates for Burgers’ equation

**Fig. 5:**
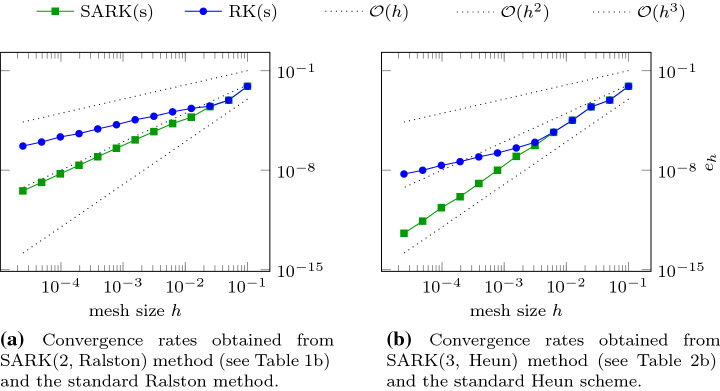
Plots of the error $e_h$ defined in (15c) for SARK and RK methods applied to the Burgers’ example described in (15)

Let us begin by returning to the one-dimensional model problem of Sect. 3 to show that the SARK methods do *not* suffer from the previously described convergence order reduction. For this discussion, the equation and error $e_h$ are as in (15). We apply Algorithm 1 with SARK schemes of $s=2$ and $s=3$ stages, collect values of $e_h$ for various *h* and plot them in Fig. 5.

The data shown in Fig. 5 was generated with the polynomial order $p=2$ in space and $h=2^{-i} / 10$ for $i=0\dots 12$. The tents were built so that (2) is satisfied with $c_{\max }=2$. Algorithm 1 is applied with $r=4$ and $r=10$ substeps within each tent for $s=2$ and $s=3$ respectively. As *h* decreases, in Fig. 5a we eventually see quadratic convergence for the two-stage SARK method (although the convergence rate seems to be slightly higher in a preasymptotic regime), while the rate of the underlying standard Runge–Kutta method drops to first order. The three-stage SARK method in Fig. 5b shows cubic convergence while the rate of the underlying standard Runge–Kutta method drops to first order again. These plots clearly show the benefit of using SARK scheme over the corresponding standard Runge–Kutta scheme.

### Convergence rates for a 2D Euler system

Now we apply SARK methods to the Euler system. Similar to the Burgers’ example, which we discussed in the previous section, we choose smooth initial data and fix a final time before the onset of shock so that no limiting is needed.

Recall that the Euler system fits into (3) with

37a $$\begin{aligned} u = \begin{pmatrix} \rho \\ m \\ E \end{pmatrix}, \quad g(u) = u, \quad f(u) = \begin{pmatrix} m \\ m\otimes m/\rho + {\scriptstyle {P}}I \\ ( E + {\scriptstyle {P}}) m / \rho \end{pmatrix}. \end{aligned}$$

Here the functions $\rho :\Omega _0\rightarrow {\mathbb {R}}$, $m:\Omega _0\rightarrow {\mathbb {R}}^2$ and $E:\Omega _0\rightarrow {\mathbb {R}}$ denote the density, momentum, and total energy of a perfect gas in the spatial domain $\Omega _0=[0,1]^2$. Furthermore, we use ${\scriptstyle {P}}=\tfrac{1}{2}\rho {\scriptstyle {T}}$ for the pressure, ${\scriptstyle {T}}= \tfrac{4}{d}\big ( \tfrac{E}{\rho }-\tfrac{1}{2}\tfrac{|m|^2}{\rho ^2}\big )$ for the temperature and $d=5$ denotes the degrees of freedom of the gas particles. The initial values are set by

37b $$\begin{aligned} \rho _0&= 1+e^{-100((x-0.5)^2+(y-0.5)^2)}, \end{aligned}$$

37c $$\begin{aligned} m_0&= (0,0)^\top , \end{aligned}$$

37d $$\begin{aligned} {\scriptstyle {P}}_0&= 1+e^{-100((x-0.5)^2+(y-0.5)^2)}, \end{aligned}$$

and the final time $t_{\max }=0.1$.

**Fig. 6:**
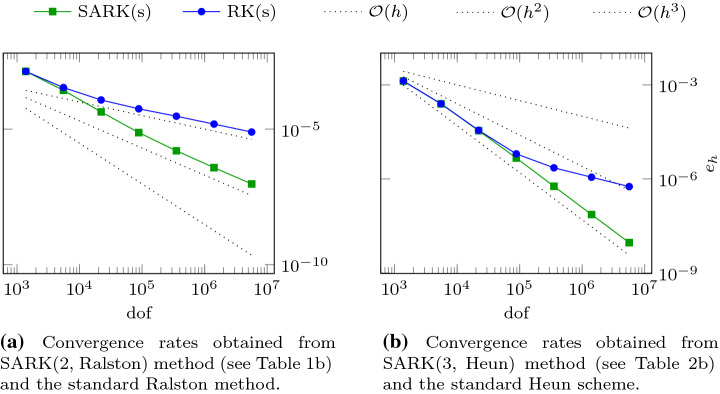
Error $e_h$ as defined in (37e) over spatial degrees of freedom (dof) for SARK and standard RK methods applied to the Euler equation on tents as described in (37)

The data shown in Fig. 6 was generated with polynomial degree $p=2$ in space and mesh sizes $h=0.1\times 2^{-i}$, for $i=0\dots 6$. For the tent generation $c_{\max }$ in (2) was set to 8 and the number of substeps $r=4$. Since we do not have an exact solution in closed form, we compare the numerical solution computed using $c_{\max }$ with a “reference solution” computed with the higher characteristic speed $2\cdot c_{\max }$. The latter requires many more tents to reach the final time. Let the former and latter approximations to $u(\cdot , t_{\max })$ be denoted by $u_h$ and $u_h^{\text {ref}}$, respectively. We define the error by

37e $$\begin{aligned} {e_h}:=\left\| u_h - u_h^{\text {ref}}\right\| _{L^2(\varOmega _0)}. \end{aligned}$$

This is the quantity that is plotted in Fig. 6.

The errors of the two-stage SARK method and the underlying RK method is seen to diverge already for the first refinement level in Fig. 6a. While the SARK method shows the expected second convergence order, the rate of the RK method drops to first order. For the three-stage methods in Fig. 5b, we see cubic convergence for both method for the first few refinements. The convergence rate of the RK method eventually drops to first order while the SARK converges at third order.

### Mach 3 wind tunnel

We conclude with the well-known benchmark example [14] of the wind tunnel with a forward-facing step onto which gas flows at Mach 3. The situation is modeled by the already described Euler system (37a), but now with the initial values

38 $$\begin{aligned} \rho _0 = 1.4, \quad m_0 = \rho _0(3,0)^\top , \quad {\scriptstyle {P}}_0 = 1 \end{aligned}$$

on a spatial domain $\Omega _0$ with a re-entrant corner at the edge of the forward-facing step — the domain and the boundary conditions are exactly as illustrated in numerous previous works, see e.g., [5, Fig. 4(a)]. Our numerical experience with this problem shows that it is beneficial to use high order local time stepping. As in our prior study [5], we use a spatially refined mesh near the re-entrant corner and let the tents adapt, providing automatic local time stepping. In contrast to the standard time stepping used in Ref. [5], we now use one of the newly proposed SARK schemes.

We shall apply the SARK(3, Heun) method. Unlike the study in Sect. 7.2, now we must handle multiple shocks that develop over time, so it is necessary to add some stabilization to the system. This is done by adding artificial viscosity based on the entropy residual as suggested by [7]—details of this stabilization on tents are exactly as already described in Ref. [5], so we omit them here.

**Fig. 7:**
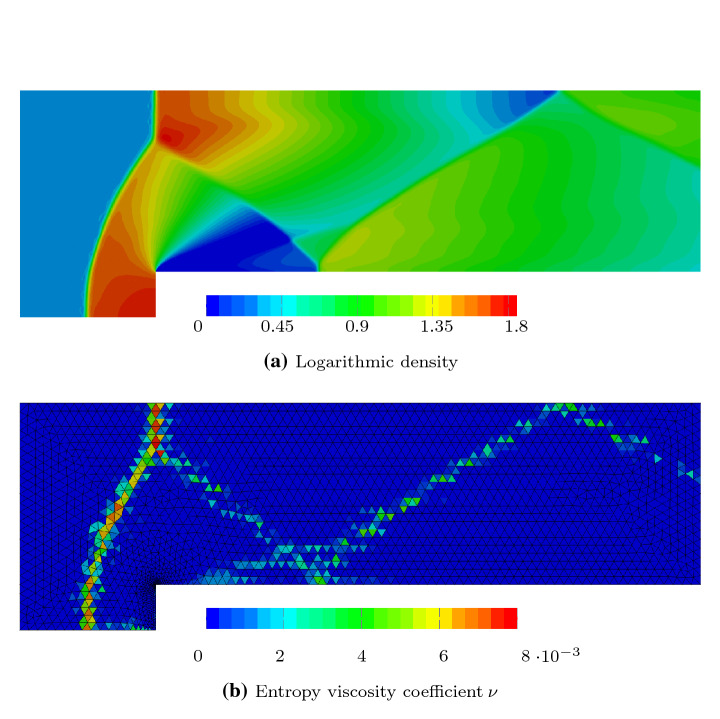
Solution of the Mach 3 wind tunnel with a forward-facing step at the final time $t_{\max }=4$ solved on 4128 triangles with SARK(3, Heun) and spatial degree $p=4.$

**Fig. 8:**
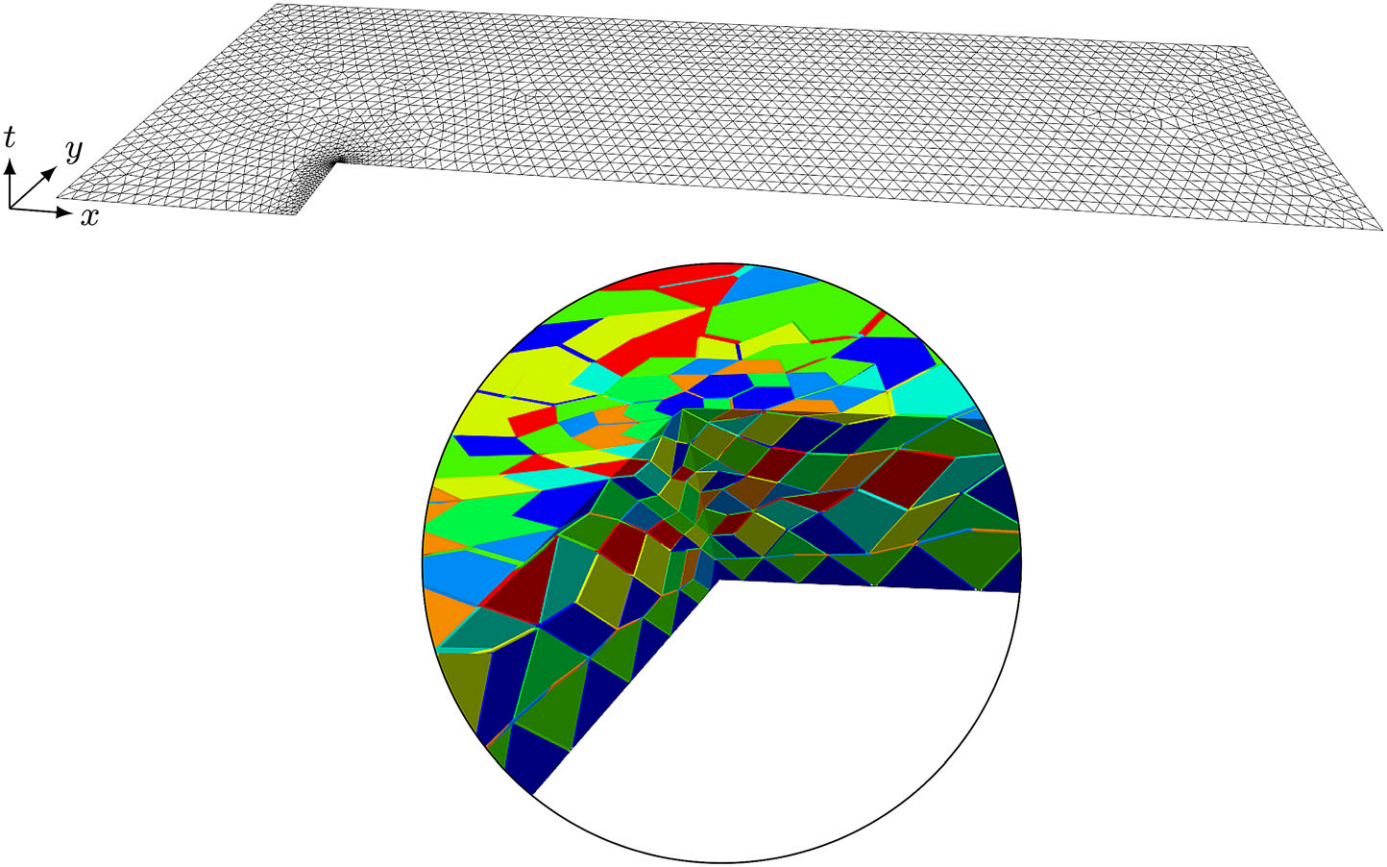
Locally refined spatial mesh (top) used for the Mach 3 wind tunnel example and a zoomed in view of the spacetime tents at the refined corner showing the automatic local timestepping. (In the spacetime figure, vertical direction represents time)

One of the components of the computed solution is shown in Fig. 7. This was generated with polynomial order $p=4$ in space, maximal characteristic speed $c_{\max }=10$ and $r=16$ substeps within each tent. Figure 8 shows the spatial mesh with the locally refined corner. The zoom in illustrates the local refinement of the tents which comes in naturally through the causality constraint while pitching the tents. The solution component (logarithmic density) shown in Fig. 7a is comparable with the solution we previously obtained using standard methods in Ref. [5], but now due to the higher accuracy of the new SARK time integration, we obtained a similar quality solution faster (with the overall simulation time on the same processor reduced by a factor of 10). We also observed that the entropy residuals calculated off the computed solution with SARK schemes led to a significantly reduced addition of artificial viscosity. The artificial viscosity coefficients generated by the entropy residual are shown in Fig. 7b, which is about half the size of what is shown in the corresponding plot in our earlier work [5, Fig. 5].
